# Production of *Pseudomonas aeruginosa* Intercellular Small Signaling Molecules in Human Burn Wounds

**DOI:** 10.4061/2011/549302

**Published:** 2011-03-17

**Authors:** Yok-Ai Que, Ronen Hazan, Colleen M. Ryan, Sylvain Milot, François Lépine, Martha Lydon, Laurence G. Rahme

**Affiliations:** ^1^Department of Surgery, Harvard Medical School and Massachusetts General Hospital, Boston, MA 02114, USA; ^2^Department of Microbiology and Molecular Genetics, Harvard Medical School, Boston, MA 02115, USA; ^3^Shriners Hospitals for Children Boston, Boston, MA 02114, USA; ^4^INRS-Institut Armand-Frappier, Laval, QC, Canada H7V 1B7

## Abstract

*Pseudomonas aeruginosa* has developed a complex cell-to-cell communication system that relies on low-molecular weight excreted molecules to control the production of its virulence factors. We previously characterized the transcriptional regulator MvfR, that controls a major network of acute virulence functions in *P. aeruginosa* through the control of its ligands, the 4-hydroxy-2-alkylquinolines (HAQs)—4-hydroxy-2-heptylquinoline (HHQ) and 3,4-dihydroxy-2-heptylquinoline (PQS). Though HHQ and PQS are produced in infected animals, their ratios differ from those in bacterial cultures. Because these molecules are critical for the potency of activation of acute virulence functions, here we investigated whether they are also produced during human *P. aeruginosa* acute wound infection and whether their ratio is similar to that observed in *P. aeruginosa*-infected mice. We found that a clinically relevant *P. aeruginosa* isolate produced detectable levels of HAQs with ratios of HHQ and PQS that were similar to those produced in burned and infected animals, and not resembling ratios in bacterial cultures. These molecules could be isolated from wound tissue as well as from drainage liquid. These results demonstrate for the first time that HAQs can be isolated and quantified from acute human wound infection sites and validate the relevance of previous studies conducted in mammalian models of infection.

## 1. Introduction

Emerging multi-drug resistant organisms are major contributors to morbidity and mortality following burn injury [1]. *Pseudomonas aeruginosa* infections are common following burns, often presenting as wound infections [2–4]. Current prophylactic measures intended to prevent these infections in burn patients consist of isolation and use of strict sterile techniques combined with rapid surgical wound closure. Although these prevention efforts can be effective under ideal circumstances, they often fail due to insufficient nursing, anesthesia, and critical care management resources, insufficient donor sites for immediate wound closure, or spread of infection from the surrounding environment during the initial first aid provided [5]. Treatment of *Pseudomonas* wound infections, along with the frequently associated shock and respiratory failure, can involve complex fluid and ventilator management, definitive surgical debridement, and advanced wound closure technologies. The use of antimicrobial agents such as colistin (polymyxin E), tailored to navigate the most recent antibiotic resistance pattern, has been useful.

Clearly, new innovations for the prevention and treatment of *Pseudomonas* wound infections in burn patients are needed. Ongoing basic research efforts have made strides in dissecting the pathogenesis of *Pseudomonas* infections using various models of infections in plants, flies, and mice [6–10]. *P. aeruginosa* coordinates the expression of multiple virulence factors using a complex regulatory communication system, namely, Quorum Sensing (QS), which has been shown to control approximately 10% of *P. aeruginosa* genes [11, 12]. QS is a cell-density-dependent phenomenon based on the release of low-molecular weight molecules that coordinate gene expression in a cell population [11, 13, 14]. In response to population density, *P. aeruginosa* produces and secretes such molecules, some of which act as specific chemical signals that control the production of virulence factors mediating acute infection. 

A decade ago, we identified the QS transcriptional regulator MvfR (multiple virulence factors regulator) [9]. The MvfR protein controls the expression of most of *P. aeruginosa*'s acute virulence-associated factors [15] as well as the biosynthesis of more than 60 excreted anthranilic acid (AA) low-molecular weight derivatives, most of which belong to the 4-hydroxy-2-alkylquinolines (HAQs) family [12, 13, 16]. HAQ expression is subject to positive autoregulation, with two HAQs, namely, 4-hydroxy-2-heptylquinoline (HHQ) [17] and 3,4-dihydroxy-2-heptylquinoline (PQS) [18], functioning as MvfR ligands. These HAQs, in concert with MvfR, positively regulate the transcription of the *pqsABCD* [19] and *phnAB* [9] operons, which encode HAQ biosynthetic enzymes of these molecules [9, 15, 19]. PQS and HHQ are produced *in vitro* and *in vivo* in acute models of infection [9, 11, 15, 19]. We showed previously that PQS is more potent than HHQ in inducing MvfR binding to the *pqsA* promoter as well as inducing *pqsA-E* transcription in *pqsA::pqsH P. aeruginosa* mutant [19]. The relative potency of HHQ versus PQS in inducing MvfR ligand-binding domain precipitation suggests that these molecules induce different MvfR conformational changes; a difference in conformation effects could subsequently results in different binding affinities to the same MvfR target promoters, and differences in MvfR-regulated gene transcription. 

Because HAQs govern and dictate infection course, they may be of use as indicators of infection stage. Moreover, interfering with these molecules could be a means by which to control *Pseudomonas* infections. The production of *Pseudomonas* HAQs has yet to be studied in human wounds. Therefore, here, we examined for the first time whether clinically important *Pseudomonas *specimens, derived from an active burn wound infection in a human patient, produce and excrete detectable levels HAQs *in situ.*

## 2. Materials and Methods

### 2.1. Human Samples

Permission for use of excess tissue and wound drainage material for the purposes of this study and permission for medical record review were obtained from the hospital institutional review board. The samples were obtained from a discarded resected infected burn wound on the patient right leg. Three types of specimen were collected: (i) one containing necrotic muscle with fat; (ii) one wet and oozing sample with pus and liquefied fat mixed together with some saline solution from the operative field; finally (iii) drainage liquid collected out from the wound.

#### 2.1.1. Histology

Wound biopsies were performed for the purposes of diagnosis and therapy at the time of surgery in the course of the patient's treatment. The biopsy specimens were fixed in formalin, blocked in paraffin, and stained with hematoxylin and eosin. Wound drainage and resected tissues in excess of the amount required for the biopsy were used in this analysis.

#### 2.1.2. HAQ Determination

The analyzed samples were obtained from excess material. Tissues were weighed, cut into small pieces, and then homogenized in 50% methanol. Whenever appropriate, liquid was mixed 1 : 1 with 50% methanol. The quantification of HAQs was performed by liquid chromatography/mass spectrometry (LC/MS) as described elsewhere [20], using HHQ-D4 and PQS-D4 as internal standards. Positive electrospray was employed in MRM mode with 2 × 10^−3^ mTorr argon and as the collision gas and 30 V of energy to quantify HAQs by LC/MS. The following ion transitions were used: HHQ 244>159, HHQ-D4 248>163, HQNO 260>159, PQS 260>175, and PQS-D4 264>179.

## 3. Results and Discussion

### 3.1. Case Report

A 17-year-old previously healthy male touched a high voltage wire in Honduras. He sustained burns to 58% of his total body surface area (TBSA), including partial thickness burns to his face, both arms, back, and buttocks, with full thickness injury to his right lower leg and medial left thigh. His initial treatment at two different hospitals in Honduras included right lower extremity fasciotomies and multiple dressing changes. He received clindamycin, meropenem, and amikacin for an unknown period of time. He was transferred to the Shriners Boston Burns Hospital 9 days after sustaining the original injury. 

**Figure 1:**
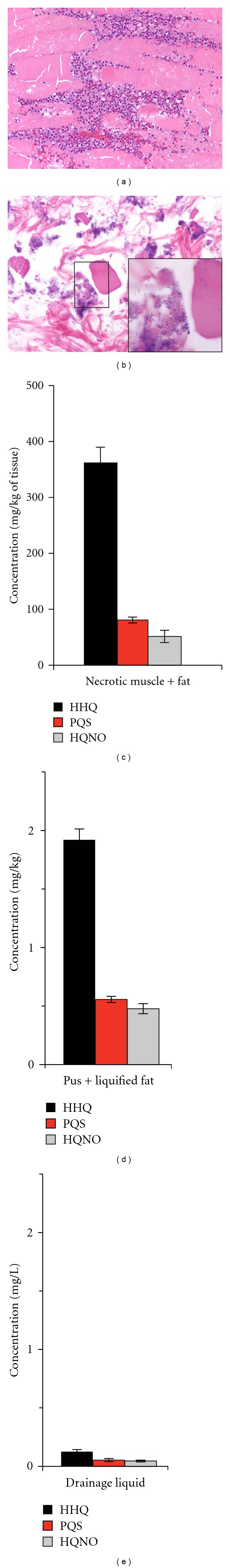
High levels of HAQs are produced in *P. aeruginosa* infected burn wounds. (a) Hematoxylin and eosin stained sections of right lower extremity biopsy specimen demonstrating active deep infection with myonecrosis and inflammatory infiltrate (10x) (b) Oil immersion view of the same specimen demonstrating myonecrosis with proliferating rods. Note the presence of intramuscular bacteria (inset). HHQ, PQS and HQNO levels measured in two operative specimens, necrotic muscle with fat (c) and pus and liquefied fat (d); and from the collected drainage liquid (e) obtained from an infected burn wound on the patient's right leg. Notably, HAQs were detected in the drainage liquid at a magnitude similar to that encountered in bacterial broth cultures.

Upon admission to Shriners Hospital, the patient was awake and alert but mildly febrile and tachycardic. There was a strong odor from his dressings. He was taken immediately to the operating room for debridement and stabilization. His lower extremity wounds were grossly infected with deep necrotic wounds to the right lower extremity where eschar had been partially resected. Biopsies showed diffuse inflammatory infiltrate and intramuscular rod-shaped bacteria (Figure 1(a)). Cultures from the right lower extremity revealed three *P. aeruginosa isolates*, a pan-resistant *Acinetobacter baumannii*, *Klebsiella pneumoniae*, *Clostridium* species, and *Candida krusei*. Initially, the *Pseudomonas* was susceptible to aztreonam, ciprofloxacin, levofloxacin, piperacillin/sulbactam, colistin; and it was resistant to amikacin, cefepime, gentamycin, meropenem, ticarcillin, tobramycin, and imipenem. *Pseudomonas* specimens from the central line tip that the patient came in with were also grown in culture. The patient was treated initially with vancomycin and meropenem as well as topical mafenide acetate. He underwent serial operative debridements over the next month. The wounds were eventually closed with autografts. He was discharged after a 2.5-month-long hospitalization.

### 3.2. Assessment of HAQs

*P. aeruginosa* acute burn wound infection is characterized by the presence of a diffuse inflammatory infiltrate related to muscle necrosis (Figure 1(a)), together with intracellular rode shaped bacteria (Figure 1(b) + inset). Bacteria-induced muscle necrosis and the bacteria's ability to invade muscle cells are two phenotypic characteristics that strongly rely on the coordinated expression of several bacterial virulence factors, many of which are under the control of MvfR [17]. We thus sought to determine the major MvfR ligands that are produced during burn wound infection in humans. As shown in Figures 1(c) and 1(d), we detected HHQ, PQS, and HQNO in whole muscle tissue as well as in drainage liquid. Interestingly, HHQ levels were higher than PQS levels. This result corroborates the ratios of these molecules that we measured previously in muscle biopsies from burned and *P. aeruginosa* infected mice [17], and are in sharp contrast to ratios measured in bacterial cell cultures [12, 13, 21], indicating that the “*in vitro” *bacterial culture setting does not reflect accurately the *in vivo* burn wound situation. Thus, in agreement with our previous results in burned and infected animals [17], we found that HHQ is produced at high levels in human burn wounds and not fully converted into PQS. PQS is converted from its precursor HHQ by the enzyme PqsH [22], whose activity is regulated by the homoserine lactone regulator LasR [22]. The low PQS levels observed in both mouse and human infected tissues might be due to reduced PqsH activity *in vivo*. Nevertheless, we have shown that PQS is fully dispensable in that *pqsH*-mutant cells, which produce HHQ but completely lack PQS, display normal MvfR-dependent gene expression and virulence in burned and infected mice [17]. Conversely, PQS is required for full production of the phenazine pyocyanin [17].

## 4. Conclusions

The findings presented here show for the first time that HAQs are produced during acute *P. aeruginosa* infections in human burn wounds. Moreover, these findings demonstrate that HAQs cell-to-cell communication molecules can be measured directly from an infected patient and that the ratio between the various HAQ molecule types resembles that found in burned and infected mice but not that produced in bacterial cell cultures. These results validate the use of burn mouse models in studies of QS-dependent *P. aeruginosa* acute infection. The fact that these QS low-molecular-weight molecules are detected also in drainage liquid collected from surgical sites is of the utmost importance for several reasons: (i) it demonstrates that these small molecules can affect large area of wounds and that in humans the bacteria can communicate beyond their immediate surroundings; (ii) it indicates that these molecules might affect other pathogens in polymicrobial infections [23, 24], such as in the presently reported case; (iii) it supports the hypothesis that targeting virulence and QS with selective anti-QS inhibitors may diminish infections caused by multi-drug resistant bacteria [10, 25].

The ability to accurately follow bacterial pathogenesis is an important prerequisite for the development of antivirulence agents aimed at controlling the course of infection. In this context, QS is a suitable drug target for new antimicrobials because it controls bacterial pathogenesis and it is evolutionarily conserved among pathogenic bacteria [26, 27]. Conventional antibiotics target a narrow spectrum of activities that are essential for bacterial growth. Pharmacological targeting of nonessential functions such as virulence factor production may decrease the emergence of resistance since bacterial survival is not directly challenged or subjected to selection pressure. For instance, we showed that pharmacologic inhibition of the MvfR regulon greatly reduced *P. aeruginosa* virulence in mice [10]. 

In conclusion, here we demonstrated that QS signaling molecules can be measured directly from an infected patient and that the ratio between the various HAQ molecule types resembles that found in burned and infected mice, demonstrating the importance of animal models in the evaluation of the efficacy of new drugs. These results also strengthen the validity of the hypothesis that QS inhibition may be an excellent target to prevent or halt multi-drug resistant *P. aeruginosa* infections.
